# Virological and serological outcomes in people with HIV-HBV coinfection who had discontinued tenofovir-containing antiretroviral therapy: Results from a prospective cohort study

**DOI:** 10.1016/j.jve.2024.100574

**Published:** 2024-12-09

**Authors:** Amir M. Mohareb, Patrick Miailhes, Julie Bottero, Caroline Lascoux-Combe, Julie Chas, Sarah Maylin, Audrey Gabassi, Hayette Rougier, Emily P. Hyle, Constance Delaugerre, Karine Lacombe, Anders Boyd

**Affiliations:** a Medical Practice Evaluation Center, Massachusetts General Hospital, Boston, USA; b Division of Infectious Diseases, Massachusetts General Hospital, Boston, USA; c Harvard Medical School, Boston, USA; d Hôpital de Fleyriat, Service de Maladies Infectieuses et Tropicales, Bourg en Bresse, 01012, France; e Etablissement Public National de Santé de Fresnes, 94260, Fresnes, France; f APHP, Hôpital Bicêtre, Service de Maladies Infectieuses, 94270, Kremlin-Bicêtre, France; g APHP, Hôpital Saint-Louis, Service de Maladies Infectieuses, Paris, F75010, France; h APHP, Hôpital Tenon, Service de Maladies Infectieuses, Paris, F75020, France; i APHP, Hôpital Saint-Louis, Laboratoire de Virologie, Paris, F75010, France; j Université de Paris, INSERM U944, Institut de Recherche Saint-Louis, F75010, Paris, France; k IMEA, Institut de Médecine et d’Epidémiologie Appliquée, Paris, F75018, France; l Harvard Center for AIDS Research, Boston, USA; m Sorbonne Université, INSERM, Institut Pierre Louis d'Épidémiologie et de Santé Publique, IPLESP, Paris, F75012, France; n APHP, Hôpital Saint-Antoine, Service de Maladies Infectieuses et Tropicales, Paris, F75012, France; o Department of Infectious Diseases, Amsterdam UMC, University of Amsterdam, Meibergdreef 9, Amsterdam, the Netherlands; p Amsterdam Institute for Immunology and Infectious Diseases, Amsterdam, the Netherlands; q Stichting hiv monitoring, Amsterdam, the Netherlands

**Keywords:** Hepatitis B, HIV, Virus replication, Tenofovir, Treatment simplification

## Abstract

**Background and Aims:**

Given advances in antiretroviral therapy (ART), some people with HIV are transitioned to non-tenofovir-containing ART; the implications for people with HIV-hepatitis B virus (HBV) are unknown. We characterized HBV-related outcomes in people with HIV-HBV coinfection while not taking tenofovir-containing ART.

**Methods:**

We analyzed participants from the French HIV-HBV Cohort Study in three treatment groups: (1) continuous tenofovir; (2) discontinued tenofovir; (3) never initiated tenofovir. We examined virological and clinical characteristics during follow-up. We assessed determinants of HBV DNA >2000 IU/mL and alanine aminotransferase (ALT) >2x upper limit of normal separately while participants were off tenofovir using univariable logistic regression with generalized estimating equations.

**Results:**

Among 192 participants, 161 (83.9 %) were on continuous tenofovir, 22 (11.5 %) discontinued tenofovir, and 9 (4.7 %) never initiated tenofovir during a median follow-up of 14.5 years (IQR = 10.5–14.8). The median proportion of within-participant visits with undetectable HBV DNA was 96.0 % (IQR = 75.0–100) in the continuous group, 100 % (IQR = 84.0–100) in the discontinued tenofovir group (while off tenofovir), and 100 % (IQR = 95.2–100) in the never initiated tenofovir group. Determinants of HBV DNA >2000 IU/mL while people were off tenofovir were detectable HIV RNA (*p* = 0.041), lower CD4^+^ T-cell count (*p* = 0.027), HBeAg positive serology (*p* = 0.004) and positive hepatitis D serology (*p* = 0.001). ALT elevation was associated with positive hepatitis C antibody serology (*p* = 0.012).

**Conclusions:**

This proof-of-concept study shows that selected people with HIV-HBV coinfection may not lose virologic control of HBV when off tenofovir. HBV virologic activity while off tenofovir may be more closely associated with uncontrolled HIV infection and positive HBeAg serology.

## 1 Introduction

More than 3 million people around the world have HIV-hepatitis B virus (HBV) coinfection.^1^ During the natural history of infection, people with HIV-HBV coinfection have a higher risk of accelerated liver damage and higher rates of hepatocellular carcinoma (HCC) than people with either infection alone.^2^^,^^3^ HIV-HBV coinfection is also associated with higher mortality compared with HIV alone, even for people who promptly initiate antiretroviral therapy (ART) and experience adequate CD4 T cell count recovery.^4^, ^5^, ^6^ In people with HIV-HBV coinfection, HBV DNA is associated with an increased risk of HCC and overall mortality; as such, HBV DNA suppression is an important therapeutic goal.^7^^,^^8^

Drugs commonly used as ART that also have activity in suppressing HBV DNA replication include tenofovir (given as either tenofovir disoproxil fumarate or tenofovir alafenamide) and lamivudine. Lamivudine is not preferred as the sole HBV-active agent due to its low genetic barrier to resistance.^9^^,^^10^ Tenofovir, on the other hand, more potentently inhibits HBV DNA replication and is not associated with the emergence of HBV resistance mutations.^11^ Therefore, clinical practice guidelines for HIV-HBV coinfection strongly recommend initiating treatment with tenofovir-containing ART and not discontinuing tenofovir.^12^

Despite these recommendations, studies from East Asia and Europe suggest that up to 15 % of people with HIV-HBV coinfection are not treated with tenofovir-containing ART.^13^, ^14^, ^15^, ^16^ Some people who experience age-related comorbidities, particularly renal disease and mineral bone disease, may be switched off of tenofovir.^17^ There is also a growing interest in strategies of medication simplification: many people with HIV-HBV coinfection are being transitioned to two-drug regimens (e.g., dolutegravir/lamivudine or dolutegravir/rilpivirine).^18^ This practice maintains HIV viral suppression, but it has not been formally evaluated in people with HIV-HBV coinfection.^19^ The use of long-acting ART (e.g., cabotegravir-rilpiverine) is also growing in popularity; this strategy maintains HIV viral suppression but does not have activity against HBV.^20^^,^^21^

The implications of transitioning people from tenofovir-containing to non-tenofovir-containing ART on HBV viral activity are largely unknown. This is concerning given that past studies, mostly in people with advanced HIV disease, have suggested cessation of tenofovir or lamivudine to be associated with HBV DNA reactivation.^22^^,^^23^ The objective of this study is to characterize the virological and serological outcomes of people with HIV-HBV who are treated with and without tenofovir-containing ART.

## 2 Materials and methods

### 2.1 Study design and population

We analyzed participants with HIV-HBV coinfection from the French HIV-HBV Cohort Study. Briefly, this was a closed, longitudinal cohort study including 308 people with HIV and chronic HBV infection from four centers located in Paris and Lyon, France.^15^ Individuals were included if they had a reactive serological result for HIV confirmed by western blot and HBsAg positive serological results for >6 months. Participants were recruited in 2002–2003 and followed up every 6–12 months until 2017–2018. The cohort design and procedures are described elsewhere.^24^

In the present study, we included individuals who had at least two visits in the cohort study. We excluded individuals who did not have a study visit after January 1, 2009 as we assumed that tenofovir usage was more common after that time.^16^

We constructed three groups of individuals with HIV-HBV infection on the basis of their anti-HBV ART: (1) individuals who had been given tenofovir and remained on tenofovir until the end of follow-up (i.e., tenofovir-continuous); (2) individuals who initiated tenofovir, but either switched to lamivudine or no anti-HBV-containing ART until the end of follow-up (i.e., tenofovir-discontinued); (3) individuals who never received tenofovir-containing ART during follow-up (i.e., no tenofovir). All chosen treatment regimens and the decision to commence or discontinue tenofovir were at the discretion of the treating physician. We defined tenofovir-discontinuation as a minimum of two consecutive visits without tenofovir-containing ART and remaining off tenofovir until the last study visit. Any participant who temporarily discontinued tenofovir for less than 6 months was considered as belonging to the continuous-tenofovir group.

All individuals provided written informed consent to participate in the study and the protocol was approved by a Hospital Ethics Committee (Paris, France) in accordance with the Helsinki Declaration.

### 2.2 Data collection

Demographic information was collected at study inclusion. Medical history of antiretroviral and anti-HBV treatments, alcohol consumption and the presence of comorbidities, including diabetes, cardiovascular disease, renal and other liver diseases, were collected at study entry and at each follow-up visit.

Laboratory data was collected at study entry and at each follow-up visit. Commercial polymerase chain reaction (PCR)-based assays were used to quantify HBV DNA viral load (VL; COBAS AmpliPrep/COBAS TaqMan; detection limit = 12 or 38 IU/mL; COBAS Amplicor; detection limit = 60 IU/mL; Roche Diagnostic Systems, Meylan, France). We defined undetectable HBV DNA at the highest threshold (<60 IU/mL). HIV RNA was measured using a commercial PCR-based assay and CD4^+^ T-cell count using standard methods. Antibodies to hepatitis C virus (HCV) and hepatitis D virus (HDV) were measured with an ELISA-based assay; if positive, serum HCV RNA and/or HDV RNA was quantified by either commercial PCR-based assay (for HCV RNA) or in-house assay (for HDV RNA).

Liver fibrosis was assessed at each yearly interval by the FibroTest calculated from a standard battery of biochemical levels.^25^ The METAVIR equivalents of these measures, established in the HIV-HBV population, were used to grade liver fibrosis (F2: 0.48–0.58, F3: 0.59–0.73, F4: ≥0.74).^26^

### 2.3 Statistical analysis

Follow-up for all participants began at cohort inclusion and continued until the last visit, loss to follow-up, or death, whichever occurred first. We summarized the characteristics of the study population at cohort inclusion and at the last follow-up visit by anti-HBV group, using medians and interquartile ranges (IQR) for continuous variables and counts and percentages for categorical variables. For the groups in which tenofovir had been initiated, we also summarized characteristics at tenofovir initiation and, if applicable, at the visit at or directly after tenofovir discontinuation. We compared the distribution of characteristics using the Kruskal-Wallis test for continuous variables and Pearson's χ^2^ or Fisher's Exact test for categorical variables.

We then plotted individual and locally weighted scatterplot smoothing (LOESS) of median levels of HBV DNA (log_10_ IU/mL) and alanine aminotransferase (ALT) (U/L) levels during follow-up. For the groups including participants who had ever received tenofovir, we produced the same plots in function of time since tenofovir initiation (in the tenofovir-continuous group) and after tenofovir discontinuation (in the tenofovir-discontinued group).

To assess the determinants for viral activity while not on tenofovir-containing ART, we modeled elevated HBV DNA (i.e., HBV DNA >2000 IU/mL) and elevated transaminases (i.e., ALT >2-times the upper limit of normal, defined at 35 U/L) over time as separate endpoints. In this analysis, we included participants who were in the tenofovir-discontinued and no tenofovir groups and the follow-up began at the time of tenofovir-discontinuation until censoring. We used logistic regression models to estimate the univariable odds ratio (OR) of having the endpoint between levels of determinants along with their 95 % confidence interval (CI). We accounted for repeat events with the use of generalized estimating equations, which included an unstructured working matrix and robust variance estimates. We did not conduct multivariable analysis for reasons described below.

We carried out statistical analysis in STATA (v15.1, College Station, TX, USA), and significance was determined by a *p*-value <0.05.

## 3 Results

### 3.1 Description of the study population

Of the 308 participants included in the cohort, 116 were not included for the following reasons: last visit was before 2009, *n* = 101; only one follow-up visit, *n* = 8; and only had one visit after tenofovir discontinuation, *n* = 7. In total, 192 participants were analyzed, of whom 161 (83.9 %) were on continuous tenofovir-containing ART, 22 (11.5 %) discontinued tenofovir-containing ART, and 9 (4.7 %) never commenced tenofovir-containing ART during follow-up. In the tenofovir-discontinued group, 6 (27 %) had been treated with dolutegravir/lamivudine dual therapy after discontinuation and 14 (64 %) were receiving lamivudine-containing ART at their last follow-up visit. In the no-tenofovir group, 1 (11 %) had been treated with dolutegravir/lamivudine dual therapy during follow-up and 6 (67 %) were receiving lamivudine-containing ART at their last follow-up visit.

Most participants analyzed were male (*n* = 158, 82.3 %) with a median age of 40 years (IQR = 35–46 years) at inclusion. Most acquired HIV through male-to-male sexual contact (*n* = 120, 62.5 %), heterosexual contact (*n* = 55, 28.7 %), or injection drug use (*n* = 15, 7.8 %). Median duration of known HIV infection was 9.2 years (IQR = 4.0–13.5) and known HBV infection 6.8 years (IQR = 2.8–11.5) at inclusion.

Characteristics at inclusion of analyzed participants were compared between anti-HBV treatment groups in Table 1. The proportion with HCV antibody positive serology was higher in the tenofovir-discontinued group (*p* = 0.022) compared to the other groups. A smaller proportion of people in the tenofovir-continuous group had evidence of advanced fibrosis or cirrhosis at inclusion (*p* = 0.012). There were no statistically significant differences in the other characteristics between groups.

**Table 1:** Description of the study population.

Characteristics at inclusion	Anti-HBV treatment group			*p*^d^
	Tenofovir-continuous (*n* = 161)	Tenofovir-discontinued (*n* = 22)	No-tenofovir (*n* = 9)	
Male sex at birth (% male)	133 (82.6)	17 (77.3)	8 (88.9)	0.72
Age, years^a^	41 (35–46)	40 (35–43)	41 (39–48)	0.68
BMI, Kg/m^2^ (*N* = 182)^a^	22.5 (21.2–24.0)	21.7 (20.0–23.2)	21.4 (20.7–24.6)	0.22
Born in high HBV endemic (HBsAg >8 %) zone^b^	48 (29.8)	6 (27.3)	3 (33.3)	0.99
Ever having an AIDS-defining illness^b^	44 (27.3)	5 (22.7)	1 (11.1)	0.65
Known HIV infection duration, years^a^	9.2 (4.0–13.4)	11.7 (6.0–14.9)	2.3 (1.5–8.4)	0.076
Duration of prior ART, years^a^	5.6 (2.3–7.4)	5.1 (2.7–6.5)	1.6 (1.3–5.9)	0.092
CD4^+^ T-cell count, per mm^3^^a^	404 (278–554)	406 (297–546)	403 (218–492)	0.88
Nadir CD4^+^ T-cell count, per mm^3^^a^ [*N* = 177]	216 (103–324)	200 (115–252)	179 (107–205)	0.82
Known HBV infection duration, years^a^	6.7 (2.9–11.1)	7.5 (5.2–12.4)	4.1 (1.5–10.0)	0.51
Prior LAM exposure^b^	142 (88.2)	17 (77.3)	7 (77.8)	0.28
Cumulative prior LAM treatment, years^a^	49.7 (25.1–69.6)	50.6 (24.3–68.5)	22.6 (15.8–73.5)	0.65
LAM-resistant mutations^b^	25/84 (70.3)	5/9 (55.6)	-/0	0.45
*Precore* mutations^b^	46/93 (49.5)	3/11 (27.3)	1/1 (100)	0.22
HBV genotype^b^	(*n* = 94)	(*n* = 13)	(*n* = 1)	0.95
A	63 (67.0)	10 (76.9)	1 (100)	
D	8 (8.5)	1 (7.7)	0 (0)	
E	9 (9.6)	1 (7.7)	0 (0)	
G	9 (9.6)	0 (0)	(0)	
Mixed A/G, A/D	5 (5.3)	1 (7.7)	0 (0)	
HCV antibody positive^b^	8 (5.0)	5 (22.7)	0 (0)	0.022
HDV antibody positive^b^	11 (6.8)	1 (4.6)	0 (0)	0.99
Advanced liver fibrosis or cirrhosis^b^, ^c^ [*N* = 190]	38 (23.9)	12 (54.6)	3 (33.3)	0.012

Abbreviations: HBsAg, hepatitis B surface antigen; AIDS, acquired immune deficiency syndrome; ART, antiretroviral therapy; BMI, body mass index; HBV, hepatitis B virus; HCV, hepatitis C virus; HDV, hepatitis D virus; HIV, human immunodeficiency virus; LAM, lamivudine.

a Median (IQR).

b Number (%).

c METAVIR F3 or F4 equivalent stage, as determined from the FibroTest®.

d Significance was determined using Kruskal-Wallis test for continuous variables and Pearson's χ^2^ test or Fisher's exact test for categorical variables.

### 3.2 HBV activity during follow-up between anti-HBV treatment groups

The median follow-up time for all participants analyzed was 14.5 years (IQR = 10.5–14.8) and median number of visits was 25 (IQR = 23–28) with no differences between anti-HBV treatment groups (*p* = 0.16 and *p* = 0.22, respectively; Table 2). Data on viral loads, serological response, and ALT levels are summarized during follow-up in Table 2. The HBV DNA and ALT levels in the tenofovir-continuous group with respect to commencing tenofovir are given in Supplementary Fig. 1. Of note, only 3 individuals with HCV positive and HDV positive antibodies at inclusion had detectable HCV RNA and HDV RNA at their last follow-up visit, respectively.

**Table 2:** Description of HBV virological, serological, and biological markers at inclusion and end of follow-up.

Characteristics	Anti-HBV treatment group			*p*
	Tenofovir-continuous (*n* = 161)	Tenofovir-discontinued (*n* = 22)	No tenofovir (*n* = 9)	
Total follow-up, years^a^	14.5 (9.3–14.7)	14.6 (14.3–14.9)	14.6 (14.3–14.7)	0.16
Number of study visits^a^	25 (20–27)	26 (23–31)	25 (23–27)	0.22
HBV DNA				
Undetectable at inclusion^b^	50 (31.1)	9 (40.9)	9 (100.0)	<0.001
Within participant % of visits with undetectable HBV DNA^b^	76.5 (55.0–94.4)	84.4 (72.7–94.7)	100 (95.2–100.0)	0.0025
Within participant % of visits with undetectable HBV DNA on tenofovir	96.0 (75.0–100)	100 (85.7–100)	–	0.12
Within participant % of visits with undetectable HBV DNA off tenofovir	–	100 (84.0–100)	–	*ntp*
Undetectable at the last study visit^b^	151 (93.8)	19 (86.4)	8 (88.9)	0.24
HBV DNA log_10_ IU/mL at the last study visit^a^, ^c^	2.83 (2.28–3.22)	3.08 (1.86–5.42)	–	*ntp*
Serological response				
HBeAg-positive at baseline^a^	90 (55.9)	8 (36.4)	1 (11.1)	0.010
HBeAg loss during follow-up^b^^d^	58 (64.4)	7 (87.5)	1 (100)	0.51
HBeAb seroconversion during follow-up^b^^d^	9 (15.5)	2 (28.6)	1 (100)	0.076
HBsAg loss during follow-up^d^	12 (7.5)	5 (22.7)	4 (44.4)	0.001
ALT, IU/L				
Levels at inclusion^a^	40 (22–67)	47 (27–92)	39 (25–46)	0.63
2x ULN (>70 IU/L) at inclusion^a^	34 (21.8)	8 (36.4)	1 (11.1)	0.26
Maximum level during follow-up^a^	91 (53–166)	120 (56–221)	69 (48–84)	0.22
Within participant % of visits with 2x ULN^d^ (>70 IU/L)	4.2 (0–15.4)	4.4 (0–27.6)	0 (0–4.5)	0.29
Within participant % of visits with 2x ULN^d^ (>70 IU/L) on tenofovir	0 (0–15.4)	0 (0–20.0)	–	0.87
Within participant % of visits with 2x ULN^d^ (>70 IU/L) off tenofovir	–	0 (0–4.8)	–	*ntp*
Last follow-up visit^a^	28 (21–39)	24 (20–33)	31 (27–33)	0.29

Abbreviations: ALT, alanine aminotransferase; HBeAg, hepatitis B “e” antigen; HBeAb, hepatitis B “e” antibody; HBsAg, hepatitis B surface antigen; HBV, hepatitis B virus; HIV, human immunodeficiency virus; ntp, no test performed.

Significance was determined using the Kruskal-Wallis test for continuous variables and Pearson's χ^2^ test or Fisher's exact test for categorical variables.

a Median (IQR).

b Number (%).

c Among patients with detectable HBV DNA or HIV RNA.

d Among HBeAg-positive patients.

**Fig. 1:**
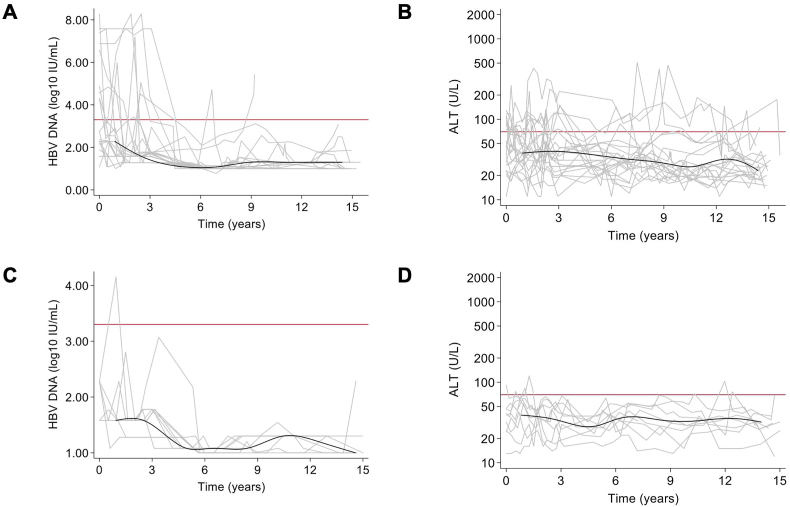
**Longitudinal follow-up of HBV markers in individuals discontinuing or without tenofovir-containing antiretroviral therapy** Description of HBV DNA and alanine aminotransferase levels during follow-up are provided in the left (**A** and **C**) and right-side (**B** and **D**) panels, respectively, for individuals who either discontinued tenofovir-containing antiretroviral therapy (**A** and **B**) or never commenced tenofovir-containing antiretroviral therapy (**C** and **D**). Average levels from a LOESS curve are given in black, while individual trajectories are given in grey lines. Red horizontal lines represent the virological and clinical thresholds used in the determinant analysis found in Table 3. Abbreviations: ALT, alanine aminotransferase; HBV, hepatitis B virus; LOESS, locally weighted scatterplot smoothing.

**Fig. 2:**
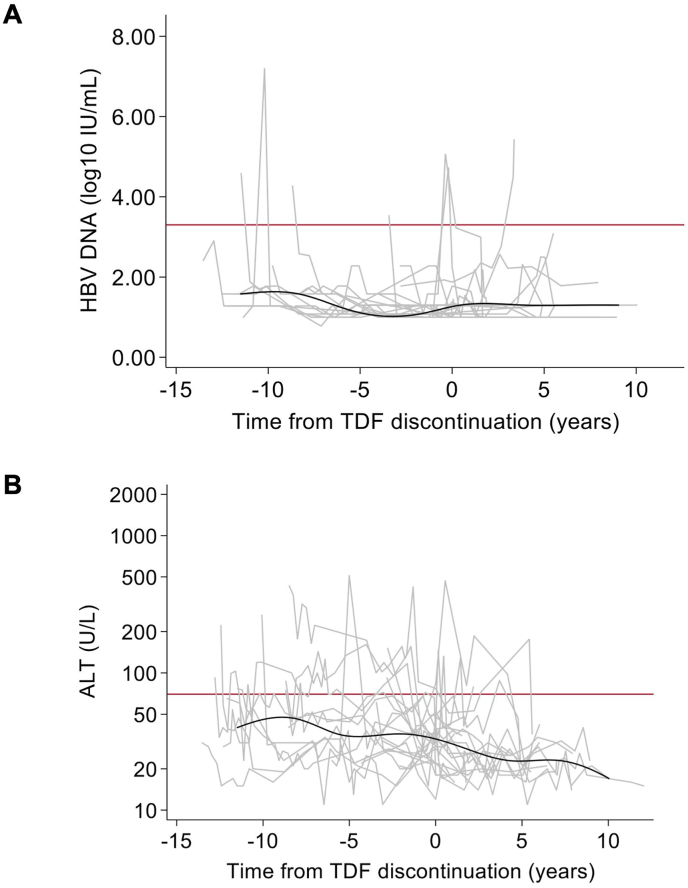
**Longitudinal follow-up of HBV markers in individuals discontinuing tenofovir-containing antiretroviral therapy** Description of HBV DNA (A) and alanine aminotransferase (B) levels over time with respect to discontinuing tenofovir-containing antiretroviral therapy are provided for individuals who discontinued tenofovir-containing antiretroviral therapy. Average levels from a LOESS curve are given in black, while individual trajectories are given in grey lines. Red horizontal lines represent the virologic and clinical thresholds used in the determinant analysis found in Table 3. Abbreviations: ALT, alanine aminotransferase; HBV, hepatitis B virus; LOESS, locally weighted scatterplot smoothing.

In the tenofovir-discontinued group, the proportion of individuals with undetectable HBV DNA was significantly higher and with ALT >2x ULN slightly lower at inclusion than in the tenofovir-continuous group (*p* < 0.001 and 0.69, respectively). As shown in Fig. 1A, HBV DNA and ALT levels slowly decreased from baseline over time. In the follow-up specifically after tenofovir discontinuation, HBV DNA and ALT levels had substantial fluctuation, yet these levels did not appear to change from the follow-up prior to discontinuation (Fig. 2A and B, respectively). At the end of follow-up, 19 (86.4 %) participants in the tenofovir-discontinued group had undetectable HBV DNA viral loads, and median ALT levels were 24 (IQR = 20–33) U/L. There was no evidence that these statistics differed from those in the tenofovir-continuous group (*p* = 0.19 and 0.15, respectively).

In the no-tenofovir group, the proportion with undetectable HBV DNA and ALT >2x ULN were slightly higher at inclusion than in the tenofovir-continuous group (*p* = 0.35 and 0.13, respectively). As shown in Fig. 1B; HBV DNA and ALT levels were controlled for the most part, but fluctuations in ALT under 2x ULN were apparent during follow-up. At the end of follow-up, 8 (88.9 %) participants in this group had undetectable HBV DNA viral loads and median ALT levels was 31 (IQR = 27–33) U/L. There was no evidence that these statistics differed from those in the tenofovir-continuous group (*p* = 0.46 and 0.60, respectively); however, this comparison involved few participants in the no-TDF group and so may not have sufficient statistical power to detect a difference.

Of note, 5 (22.7 %) and 4 (44.4 %) participants lost HBsAg by the end of follow-up in the tenofovir-discontinuation and no-tenofovir groups, respectively. These proportions were significantly higher than in the tenofovir-continuous group (*p* = 0.037 and *p* = 0.005, respectively). In the tenofovir-discontinuation group, HBsAg-loss occurred before discontinuation in 3 participants (range of follow-up prior to discontinuation 3.5–11.7) and after discontinuation in 2 participants (range of follow-up after discontinuation 3.7–5.4). In the no-tenofovir group, 2 participants lost HBsAg during treatment with lamivudine and the remaining 2 without any anti-HBV agents.

### 3.3 Determinants of virological and clinical endpoints while not on tenofovir-containing antiretroviral treatment

Median follow-up time while off tenofovir was 4.8 years (IQR = 1.8–6.4) in the tenofovir-discontinued and 14.6 years (IQR = 14.4–14.7) in the no-tenofovir group. During this follow-up, which included 480 visits with measured HBV DNA, there were 9 (1.9 %) visits at which HBV DNA was greater than 2000 IU/mL, occurring in 8 participants. Of these visits, 8 (in 7 individuals) and 1 (in 1 individual) were in the tenofovir-discontinued and no-tenofovir groups, respectively. As shown in Table 3, determinants for HBV DNA greater than 2000 IU/mL were having detectable HIV RNA (*p* = 0.041), lower CD4^+^ T-cell count (*p* = 0.027), HBeAg positive serology (*p* = 0.004) and HDV antibody positive serology (*p* = 0.001). Multivariable analysis was precluded by the low number of visits with HBV DNA greater than 2000 IU/mL.

**Table 3:** Univariable determinants of virological and clinical endpoints in individuals not receiving tenofovir-containing antiretroviral therapy.

	HBV DNA >2000 IU/mL		ALT >2x ULN	
	OR (95%CI)	*p*	OR (95%CI)	*p*
Anti-HBV treatment group				
Tenofovir-discontinued	Ref		Ref	
No tenofovir	0.27 (0.04–2.07)	0.21	0.20 (0.06–0.72)	0.014
Sex at birth				
Male	Ref		Ref	
Female	0.73 (0.10–5.10)	0.75	∗	
Age (per 5 years)	1.11 (0.58–2.12)	0.76	0.82 (0.65–1.04)	0.10
Region of HBV endemicity				
Low/moderate	Ref		Ref	
High	0.31 (0.04–2.31)	0.26	0.25 (0.05–1.33)	0.10
AIDS-defining illness^†^				
No	Ref		Ref	
Yes	2.28 (0.49–10.51)	0.29	1.66 (0.28–9.95)	0.58
Detecable HIV RNA				
>50 copies/mL	Ref		Ref	
≤50 copies/mL	0.26 (0.07–0.95)	0.041	0.66 (0.31–1.44)	0.30
CD4^+^ T-cell count (per √mm^3^)	0.88 (0.78–0.99)	0.027	0.98 (0.91–1.05)	0.60
Nadir CD4^+^ T-cell count (per √mm^3^)	0.91 (0.79–1.05)	0.21	1.05 (0.94–1.17)	0.41
HBeAg status				
Negative	Ref		Ref	
Positive	21.66 (2.64–177.76)	0.004	2.79 (0.68–11.53)	0.16
Prior LAM exposure				
No	Ref		Ref	
Yes	0.47 (0.06–3.84)	0.48	0.64 (0.32–1.29)	0.21
Anti-HCV antibody status				
Negative	Ref		Ref	
Positive	3.98 (0.73–21.79)	0.11	4.35 (1.38–13.71)	0.012
Anti-HDV antibody status				
Negative	Ref		Ref	
Positive	9.51 (2.49–36.36)	0.001	2.84 (0.93–8.66)	0.066

Abbreviations: AIDS, acquired immune deficiency syndrome; ALT, alanine aminotransferase; ART, antiretroviral therapy; HBV, hepatitis B virus; HCV, hepatitis C virus; HDV, hepatitis D virus; HIV, human immunodeficiency virus; CI, confidence interval; LAM, lamivudine; OR, odds ratio; ULN, upper limit of normal.

∗Model did not converge and thus OR could not be given.

During follow-up after tenofovir discontinuation with a total of 657 visits with ALT measurements, there were 80 (12.2 %) visits at which ALT was greater than 2x ULN, occurring in 16 participants. Of these visits, 72 (in 12 individuals) and 8 (in 4 individuals) were in the tenofovir-discontinued and no-tenofovir groups, respectively. Severe ALT flares of greater than 5x ULN were observed at 13 visits in 5 individuals. Two of these flares were likely due to active HCV replication. As shown in Table 3, determinants for ALT greater than 2x ULN were being in the tenofovir-discontinued versus no-tenofovir group (*p* = 0.014), and HCV antibody positive serology (*p* = 0.012). Again, multivariable analysis was precluded by the low number of visits with ALT greater than 2x ULN.

## 4 Discussion

We analyzed a cohort of people with HIV-HBV coinfection who were treated with and without tenofovir-containing ART and who had frequent testing of HBV DNA, HBeAg, HBsAg, and ALT levels throughout their period of observation. We found no significant virological activity or hepatitis flares in people who had either discontinued or never started tenofovir in comparison to people who had continually been treated with tenofovir. Importantly, people who had discontinued or never initiated tenofovir were more likely to lose HBsAg during follow-up in comparison with people on continuous tenofovir-containing ART. This proof-of-concept study suggests that in selected people with HIV-HBV coinfection, discontinuing tenofovir may not lead to any deleterious consequences on controlling HBV activity.

It should be immediately stressed that any comparisons between people continuously treated with tenofovir and discontinuing or never initiating tenofovir are limited by indication bias. Baseline characteristics of the different subgroups of people suggest that people with more HBV disease activity were more likely initially treated and continued on tenofovir-containing ART. Despite its lower barrier to resistance, lamivudine may be effective in maintaining viral suppression in people with HIV-HBV coinfection and low HBV viral activity.^27^^,^^28^ Studies of people with HBV monoinfection demonstrate that some people with suppressed HBV DNA can stop antiviral therapy without HBV reactivation.^29^^,^^30^ A recent case series of 19 people with HIV-HBV coinfection who switched to intermittent ART (three to five days per week) showed no HBV DNA rebound.^31^ The data presented here further suggests that HBV DNA suppression is possible in people with HIV-HBV coinfection without tenofovir, but it must be in a selected group of people with low viral activity. As the criteria of low viral activity is not fully defined, clinicians should still follow current guidance, which strongly recommend using tenofovir-containing ART for people with HIV-HBV coinfection.

An important finding in this study is the characterization of factors associated with having HBV DNA elevation and hepatic inflammation in people not receiving tenofovir. Elevations in HBV DNA (above 2000 IU/mL) more commonly occurred in people with HBeAg positive disease, detectable HIV RNA, and lower CD4 T cell counts, demonstrating that immunological control of HBV is an important predictor of activity. Given the efficacy of antiretroviral treatment during the study period, the positive association with detectable HIV RNA and elevations in HBV DNA suggests that inconsistent ART adherence was a likely contributor to this finding. Coinfection with HCV and HDV also appears to be associated with adverse virological outcomes and hepatic inflammation. Past studies have demonstrated the role these coinfections play in increasing overall mortality in people with HIV-HBV coinfection.^32^, ^33^, ^34^ Though the observational period in the present study mostly occurred prior to the availability of direct acting antivirals for HCV, there is a need for early screening and treatment for viral coinfections and monitoring for liver-related complications in people with HIV-HBV.

A novel finding from this study is the demonstration of HBsAg loss, which is considered a functional cure of HBV, with tenofovir-free regimens in HIV-HBV coinfection. Notwithstanding the limited numbers of individuals who had either discontinued or never initiated tenofovir, these had a very high proportion with HBsAg loss compared to what is expected in those who maintained tenofovir therapy.^35^ This finding could be related to baseline HBV disease activity, as people with lower HBV virologic replication and lower quantitative HBsAg levels are known to have a higher incidence of HBsAg loss.^15^^,^^35^ However, it is also likely that cessation of tenofovir stimulates the host immune response to clear HBsAg, an observation that has been studied in HBV monoinfection.^29^^,^^36^^,^^37^ One strategy for achieving HBV cure (i.e., HBsAg loss) in HBV monoinfection involves cessation of nucleotide/nucleoside analogue therapy.^38^ The results of our study suggest that in selected patients with HIV-HBV coinfection, tenofovir cessation, while remaining on effective ART, can be pursued as a possible strategy for HBV cure.

A major strength of this study is the frequent monitoring of HBV DNA, HBV serological markers, and ALT levels in this cohort of people with HIV-HBV coinfection. Nevertheless, this study had several limitations. First, only individuals who were able to continue follow-up after 2009 were included in this study, which could have selected people with more consistent clinical care. However, given the monitoring required for potential virological and biochemical rebound after tenofovir discontinuation, these individuals are more likely to be candidates for non-tenofovir containing therapies in future studies. Second, we do not have detailed information about HBV virological control or past hepatitis flares experienced prior to enrolment in this cohort, which may still impact or at least be associated with long-term virological and clinical events. Third, we do not have data on the reasons for ART decision-making so we cannot determine whether tenofovir was not used due to drug toxicity, clinician discretion, or patient preference. Fourth, the small sample size of people in non-tenofovir-containing comparator groups precluded multivariable analysis. Finally, as mentioned above, the very likely possibility of indication bias means that groups should be viewed as distinct populations rather than comparison groups.

## 5 Conclusion

In conclusion, we did not find evidence of HBV reactivation or hepatitis flares in people with HIV-HBV coinfection who had either discontinued or were never treated with tenofovir-containing ART. People who were not treated with tenofovir had a notably high proportion of HBsAg loss. This study has implications for people with HIV-HBV coinfection, particularly in light of the growing interest in ART strategies without tenofovir, including use of two-drug therapy in ART simplification and long-acting injectable ART without HBV activity. Future studies of HIV-HBV coinfection should evaluate clinical outcomes associated with tenofovir discontinuation, such as the development of fibrosis, cirrhosis, and HCC.

## CRediT authorship contribution statement

**Amir M. Mohareb:** Writing – review & editing, Writing – original draft, Conceptualization. **Patrick Miailhes:** Writing – review & editing. **Julie Bottero:** Writing – review & editing. **Caroline Lascoux-Combe:** Writing – review & editing. **Julie Chas:** Writing – review & editing. **Sarah Maylin:** Writing – review & editing, Data curation. **Audrey Gabassi:** Writing – review & editing. **Hayette Rougier:** Writing – review & editing, Project administration, Data curation. **Emily P. Hyle:** Writing – review & editing. **Constance Delaugerre:** Writing – review & editing. **Karine Lacombe:** Writing – review & editing, Funding acquisition. **Anders Boyd:** Writing – review & editing, Writing – original draft, Funding acquisition, Formal analysis, Data curation, Conceptualization.

## Ethics approval

This study was conducted in accordance with the Helsinki Declaration. The protocol was approved by a Hospital Ethics Committee (Paris, France).

## Data availability

Data are available on formal request to the principal investigator (AB). Requests for data transfer need to be approved by the Scientific Committee of the French HIV-HBV cohort. All data transfers need to be formally agreed upon per the European Union's General Data Protection Regulations.

## Funding

This work was supported by SIDACTION and ANRS. AMM is supported by the National Institute for Allergy and Infectious Diseases at the US National Institutes of Health (grant number K01AI166126) and the Harvard University Center for AIDS Research (grant number P30AI060354). EPH is supported by the Jerome and Celia Reich Endowed Scholar in HIV/AIDS Research at MGH. The funders had no role in the design or authorship of this publication. The article contents are solely the responsibility of the authors and do not necessarily represent the official views of the funders.

## Declaration of competing interest

The authors declare the following financial interests/personal relationships that may be considered as potential competing interests: Karine Lacombe reports a relationship with Gilead Sciences Inc, MSD Merck Sharp & Dohme AG, and ViiV Healthcare. Anders Boyd reports a relationship with Gilead Sciences Inc that includes speaking and lecture fees. All other authors declare that they have no known competing financial interests or personal relationships that could have appeared to influence the work reported in this paper.
