# Supplementary material for: Virological and serological outcomes in people with HIV-HBV coinfection who had discontinued tenofovir-containing antiretroviral therapy: Results from a prospective cohort study
Source: J Virus Erad. 2024 Dec 9;10(4):100574. doi: 10.1016/j.jve.2024.100574 (PMC11841080; doi:10.1016/j.jve.2024.100574)
Supplement: Multimedia component 1 [file mmc1.docx]

**SUPPLEMENTARY FIGURE**

**Supplementary Figure 1. Longitudinal follow-up of HBV markers in individuals before and after commencing tenofovir-containing antiretroviral therapy for those who remained on this anti-HBV regimen**

| **A** | **** |
| --- | --- |
| **B** | **** |

Description of HBV DNA (**A**) and alanine aminotransferase (**B**) levels over time with respect to commencing tenofovir-containing antiretroviral therapy are provided for individuals who remained on tenofovir-containing antiretroviral therapy. Average levels from a LOESS curve are given in black, while individual trajectories are given in grey lines. Red horizontal lines represent the virologic and clinical thresholds used in the determinant analysis found in Table 3.

Abbreviations: ALT, alanine aminotransferase; HBV, hepatitis B virus; LOESS, locally weighted scatterplot smoothing
